# Supplementary material for: A Feasibility Study of Functional Lung Volume Preservation during Stereotactic Body Radiotherapy Guided by Gallium-68 Perfusion PET/CT
Source: Cancers (Basel). 2023 Mar 11;15(6):1726. doi: 10.3390/cancers15061726 (PMC10046099; doi:10.3390/cancers15061726)
Supplement: Supplementary file 1 [file cancers-15-01726-s001.zip › cancers-2278076-supplementary.pdf]

Table S1 : Acceptable Spillage/Dose Conformity Guidelines

Ratio of Prescription Isodose Volume to the PTV		Ratio of 50% Isodose Volume to the PTV, $R_{50\%}$		Maximum Dose at 2 cm from PTV in any direaction as % of prescribed dose (PD) $D_{2cm}(Gy)$=%xPD		Percent of Lung receiving 20 Gy total or more, $V_{20}(\%)$		PTV Volume (cc)
Deviation		Deviation		Deviation		Deviation		
none	acceptable	none	acceptable	none	acceptable	none	acceptable	
<1.2	<1.5	<5.9	<7.5	<50.0	<57.0	<10	<15	1.8
<1.2	<1.5	<5.5	<6.5	<50.0	<57.0	<10	<15	3.8
<1.2	<1.5	<5.1	<6.0	<50.0	<58.0	<10	<15	7.4
<1.2	<1.5	<4.7	<5.8	<50.0	<58.0	<10	<15	13.2
<1.2	<1.5	<4.5	<5.5	<54.0	<63.0	<10	<15	22.0
<1.2	<1.5	<4.3	<5.3	<58.0	<68.0	<10	<15	34.0
<1.2	<1.5	<4.0	<5.0	<62.0	<77.0	<10	<15	50.0
<1.2	<1.5	<3.5	<4.8	<66.0	<86.0	<10	<15	70.0
<1.2	<1.5	<3.3	<4.4	<70.0	<89.0	<10	<15	95.0
<1.2	<1.5	<3.1	<4.0	<73.0	<91.0	<10	<15	126.0
<1.2	<1.5	<2.9	<3.7	<77.0	<94.0	<10	<15	163.0

Table S2: Dose to organ at risk and target volumes

	Anatomical Plan	Functional Plan	Difference (p-value)
	Median (range)	Median (range)	
PTV coverage	99.6 (71.8-100)	99.6 (70.2-100)	**0.01**
ITV coverage	100 (83.4-100)	100 (83.3-100)	0.53
Spinal cord (Dmax)	7.2 (1.5-22.7)	7.9 (1.5-22.2)	0.95
Left bronchi (Dmax)	5.1 (0-37.3)	4.7 (0-37.4)	0.49
Right bronchi (Dmax)	5.3 (9.8-40.0)	5.1 (0.1-40.8)	0.94
Trachea (Dmax)	5.8 (0.04-41.4)	5.0 (0.04-41.6)	0.61
Esophagus (Dmax)	8.3 (3.4-36.4)	8.4 (1.8-35.7)	0.74
Pulmonary Artery (Dmax)	6.4 (0.1-61.8)	6.1 (0.1-61.8)	0.71
Aorta (Dmax)	11.7 (3.3-62.9)	12.6 (2.7-66.2)	0.21
Brachial Plexus (Dmax)	0 (0-35.4)	0 (0-36.0)	0.61
Heart			
• Dmax	5.7 (0.2-38.9)	6.6 (0.2-40.1)	0.13
• V39Gy	0 cc (0-0)	0 cc (0-0)	NA
Chest wall			
• Dmax	49.4 (15.4-83.7)	49.8 (16.9-82.7)	0.11
• V40Gy	0.6 cc (0-37.1)	0.5 cc (0-37.2)	0.43
Ribs			
• Dmax	47.3 (13.7-80.5)	48.6 (15.3-82.0)	0.16
• V30Gy	1.8 cc (0-34.3)	2.0 cc (0-34.2)	0.26
Liver (V15Gy)	0 cc (0-53.4)	0 cc (0-59.8)	0.81
Stomach			
• Dmax	0 (0-4.8)	0 (0-4.7)	0.86
• V16.5Gy	0 cc (0-0)	0 cc (0-0)	NA

Figure S1: Distribution of absolute difference and relative difference of the mean lung dose (MLD) between anatomical planning and functional planning in the FV70% (A,B) lung and FV90% (C,D) functional volume

A.

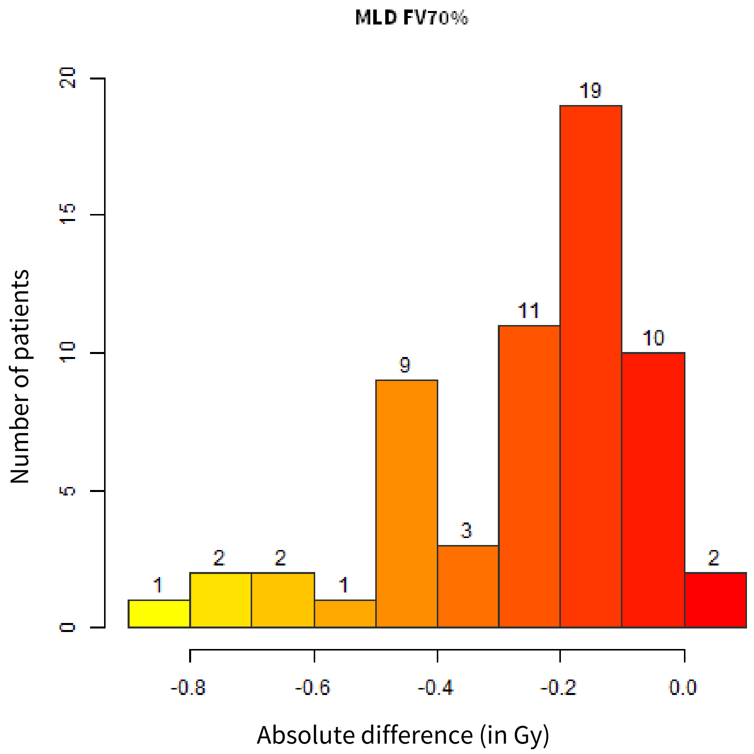

B.

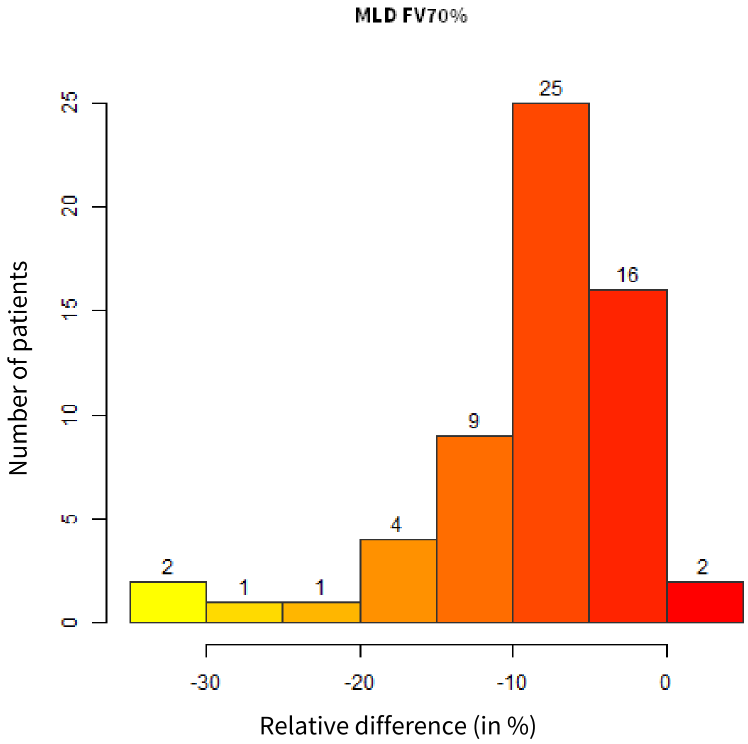

MLD FV70%
Number of patients
0
5
10
15
20
25
2
1
1
4
9
25
16
2
-30
-20
-10
0
Relative difference (in %)

C.

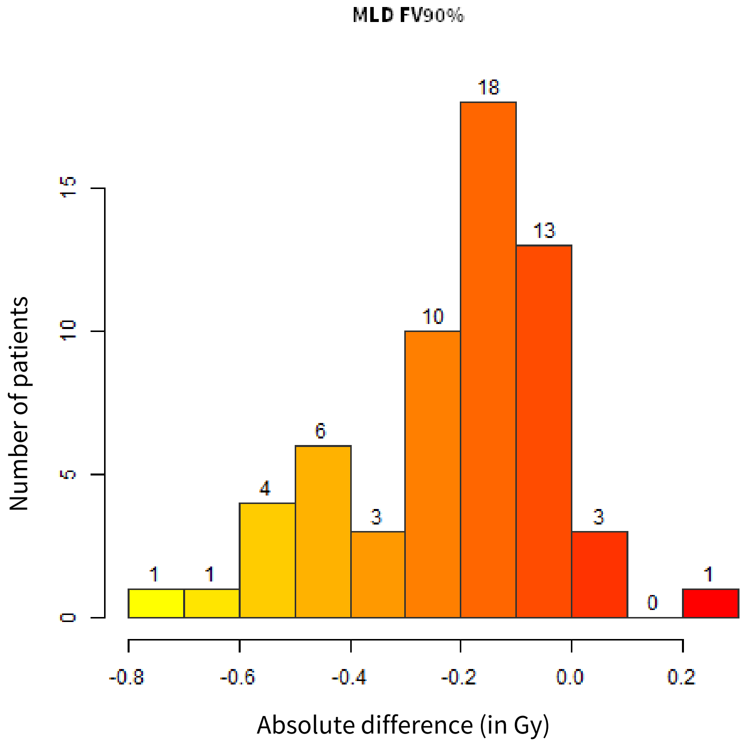

MLD FV90%
Number of patients
0
5
10
15
1
1
4
6
3
10
18
13
3
0
1
-0.8
-0.6
-0.4
-0.2
0.0
0.2
Absolute difference (in Gy)

D.

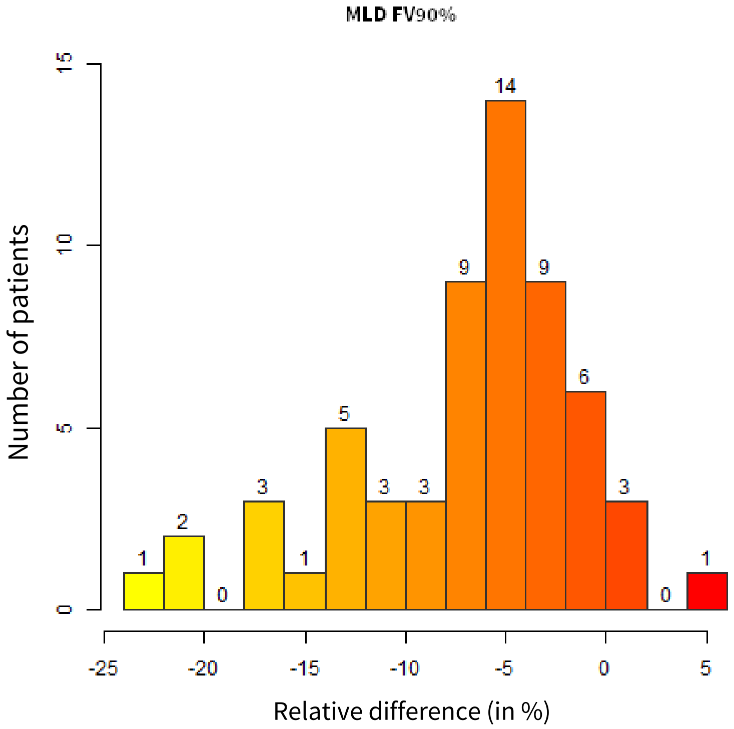

MLD FV90%
Number of patients
0
5
10
15
1
2
0
3
1
5
3
3
9
14
9
6
3
0
1
-25
-20
-15
-10
-5
0
5
Relative difference (in %)

Figure S2:
Distribution of relative difference for the V5Gy between anatomical planning and functional planning in the FV70% (A) and FV90% (B) lung functional volume

A.

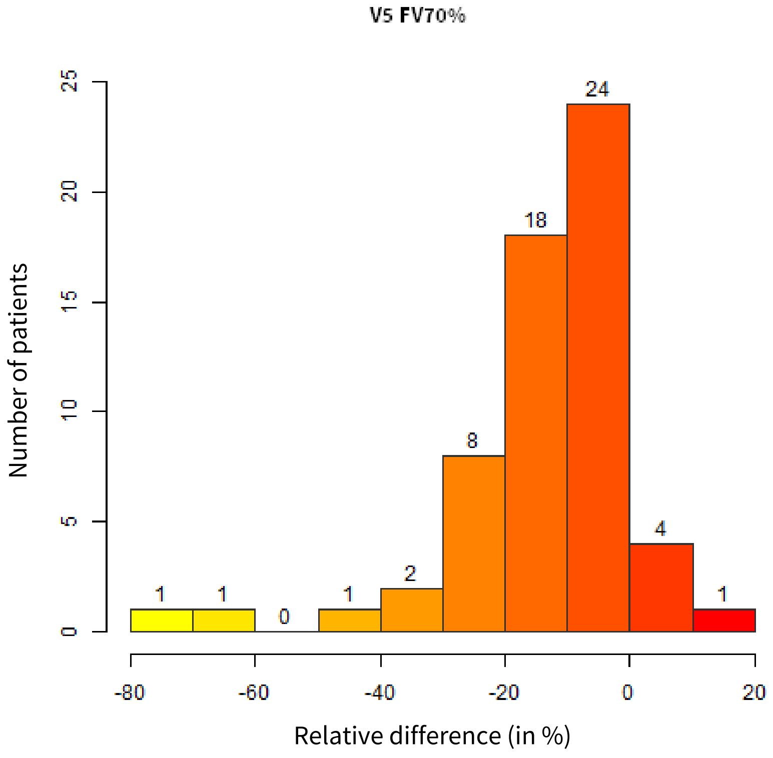

B.

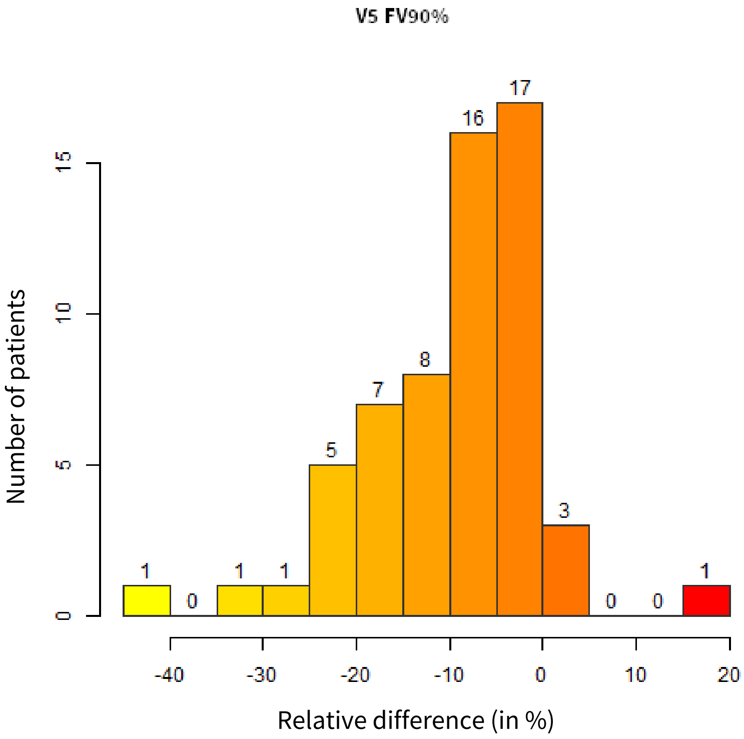
V5 FV90%
Number of patients
0
5
10
15
1
0
1
1
5
7
8
16
17
3
0
0
1
-40
-30
-20
-10
0
10
20
Relative difference (in %)

Figure S3:
Distribution of relative difference for the V10Gy between anatomical planning and functional planning in the FV50% (A), FV70% (B) and FV90% (C) lung functional volume

A.

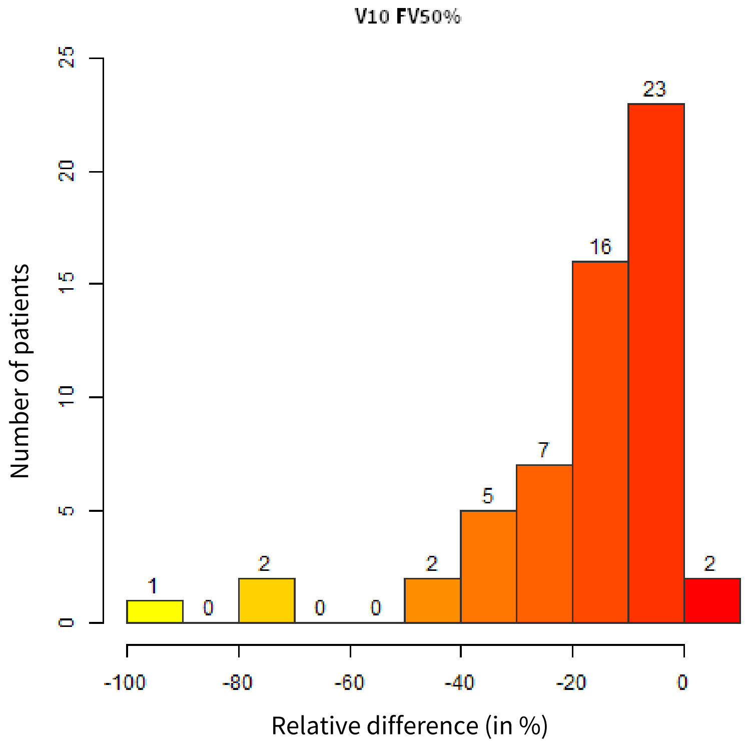

B.

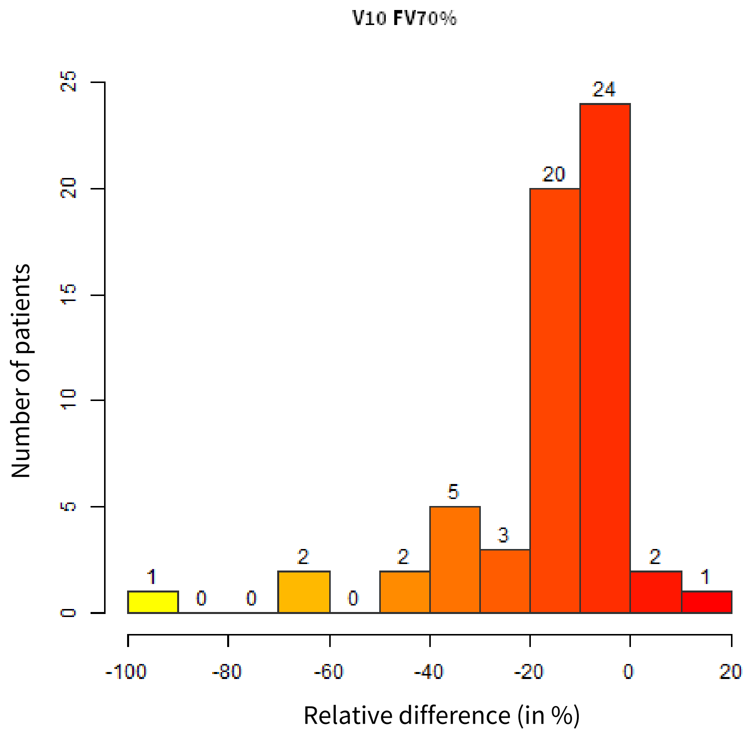
V10 FV70%
Number of patients
0
5
10
15
20
25
1
0
0
2
0
2
5
3
20
24
2
1
-100
-80
-60
-40
-20
0
20
Relative difference (in %)

C.

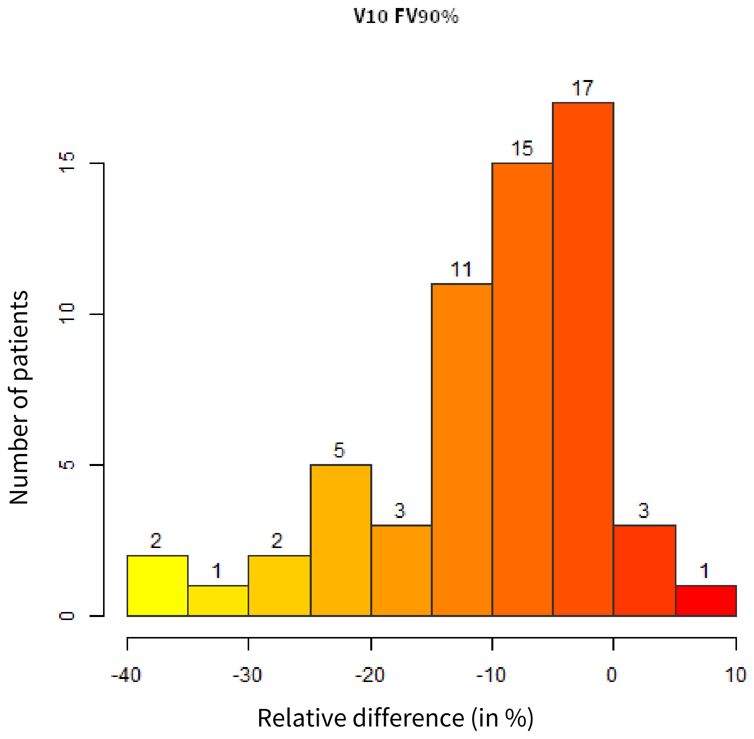
V10 FV90%
Number of patients
0
5
10
15
2
1
2
5
3
11
15
17
3
1
-40
-30
-20
-10
0
10
Relative difference (in %)

Figure S4:
Distribution of relative difference for the V15Gy between anatomical planning and functional planning in the FV50% (A), FV70% (B) and FV90% (C) lung functional volume

A.

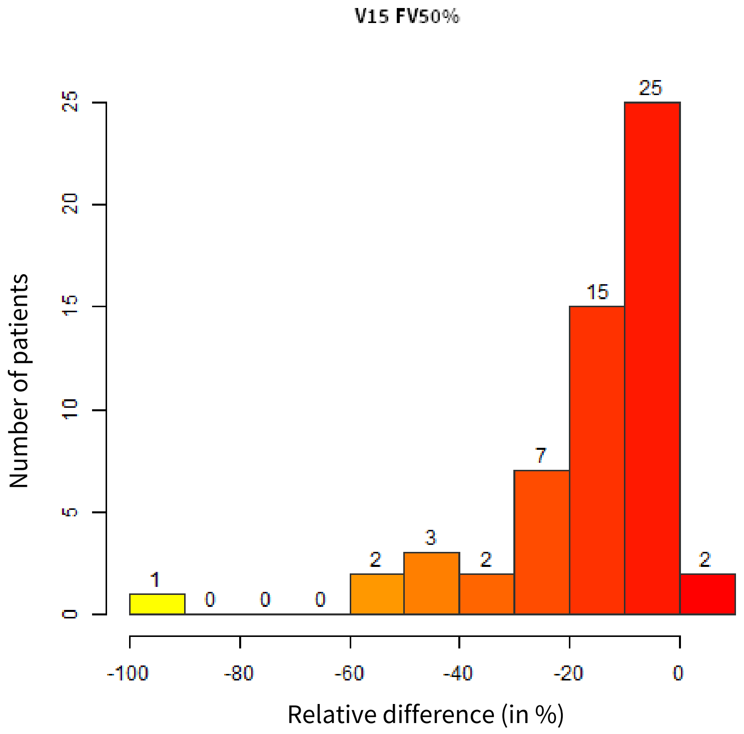

B.

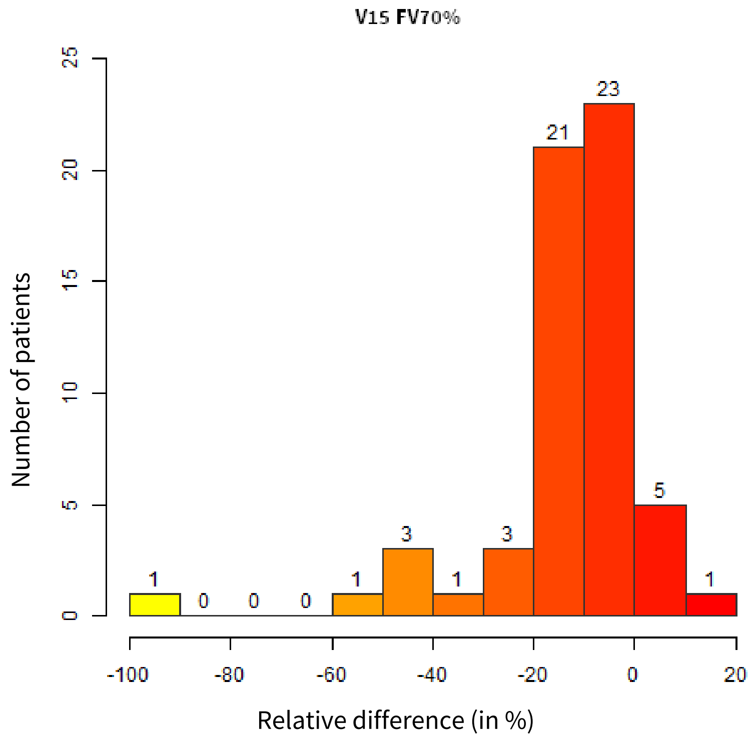

C.

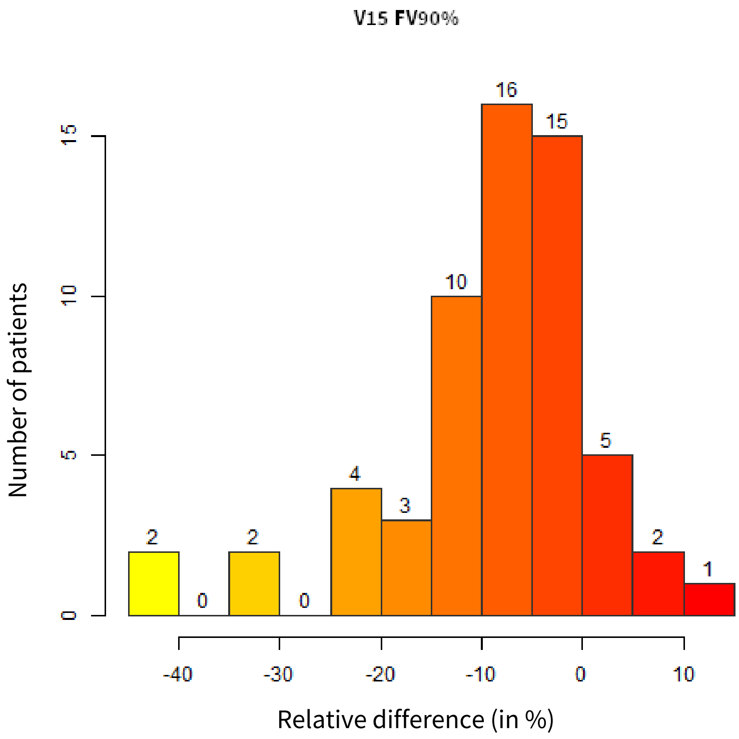
V15 FV90%
Number of patients
0
5
10
15
2
0
2
0
4
3
10
16
15
5
2
1
-40
-30
-20
-10
0
10
Relative difference (in %)

Figure S5:
Distribution of relative difference for the V20Gy between anatomical planning and functional planning in the FV50% (A), FV70% (B) and FV90% (C) lung functional volume

A.

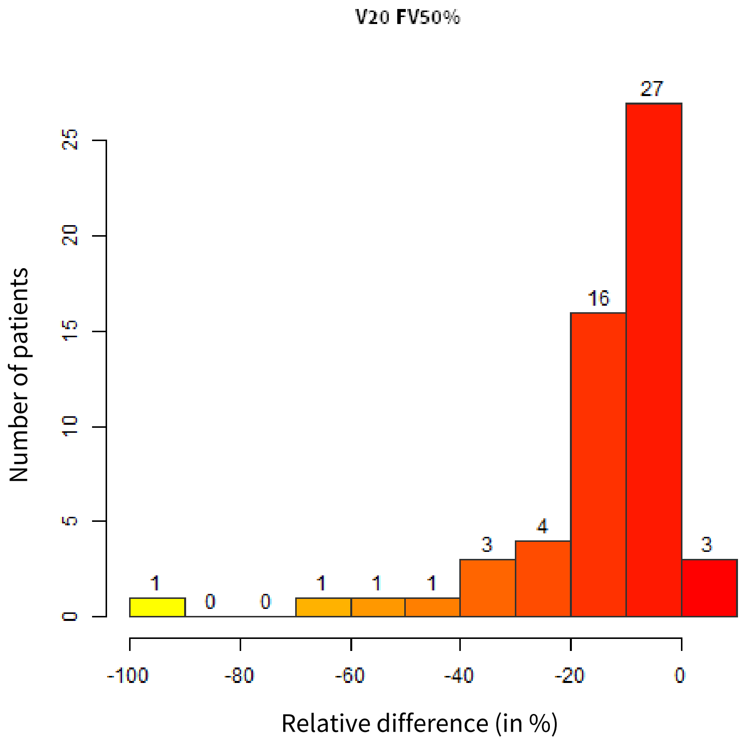

B.

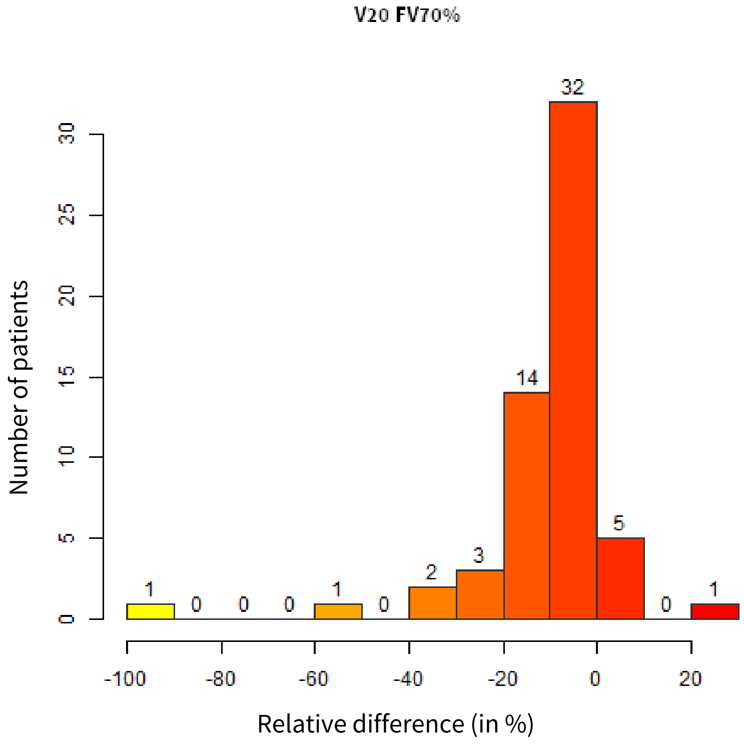

C.

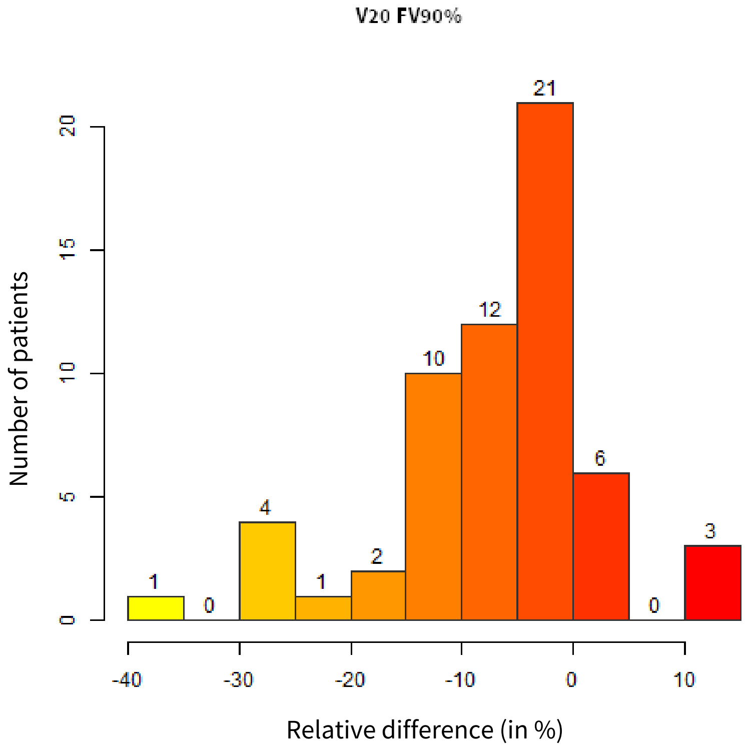
V20 FV90%
Number of patients
Relative difference (in %)
0
5
10
15
20
-40
-30
-20
-10
0
10
1
0
4
1
2
10
12
21
6
0
3
